# Adaptive law-based feature representation for time series classification

**DOI:** 10.1038/s41598-025-25667-0

**Published:** 2025-11-25

**Authors:** Marcell T. Kurbucz, Balázs Hajós, Balázs P. Halmos, Vince Á. Molnár, Antal Jakovác

**Affiliations:** 1 Institute for Global Prosperity, University College London, 9-11 Endsleigh Gardens, London, WC1H 0EH UK; 2 Faculty of Science, Eötvös Loránd University, 1/A Pázmány Péter Walkway, Budapest, 1117 Hungary; 3 Department of Computational Sciences, Wigner Research Centre for Physics, 29-33 Konkoly-Thege Miklós Street, Budapest, 1121 Hungary; 4 Faculty of Engineering and Natural Sciences, Tampere University, Kalevantie 4, 33100 Tampere, Finland; 5 Department of Statistics, Corvinus University of Budapest, 8 Fővám Square, Budapest, 1093 Hungary

**Keywords:** Time series classification, Representation learning, Feature engineering, Artificial intelligence, Computational science, Statistics

## Abstract

Time series classification (TSC) underpins applications across finance, healthcare, and environmental monitoring, yet real-world series often contain noise, local misalignment, and multiscale patterns. We introduce adaptive law-based transformation (ALT), a multiscale generalization of the earlier linear law-based transformation (LLT). ALT scans each series with variable-length, shifted windows, constructs symmetric delay embeddings, and extracts eigenvectors associated with the eigenvalue of minimal magnitude (“shapelet laws”) that capture locally stable patterns. These laws are assembled into class-specific dictionaries, and new series are projected onto them to yield compact, transparent features that enhance linear separability while remaining compatible with standard classifiers. On the BasicMotions dataset with synthetic Gaussian noise, ALT sustained test accuracy roughly 15–20 percentage points (pp) above raw inputs and 5–10 pp above LLT at moderate noise levels. Across ten datasets from the UCR Time Series Classification Archive—spanning motion, biomedical, spectroscopy, and industrial domains—ALT improved median test accuracy by + 7.6 pp with k-nearest neighbors (KNN) and + 4.8 pp with support vector machines (SVMs), with particularly large gains on long, noisy industrial series (FordA/B: + 23.1–25.3 pp). In addition, ALT often reduced SVM training time (median reductions of 340.6 s on FordB and 717.5 s on FordA) while maintaining or improving accuracy. ALT thus offers a lightweight and transparent alternative to heavyweight TSC pipelines: it requires only a small hyperparameter set, produces stable and discriminative features, and delivers competitive or superior accuracy under challenging conditions.

## Introduction

Time series classification (TSC) underpins applications in finance, healthcare, human activity recognition, remote sensing, and industrial monitoring, where the objective is to assign uni- or multivariate sequences to predefined classes^1,2^. Real-world series pose several challenges for learning algorithms: noise and non-stationarity, local temporal misalignment, class overlap, and—crucially—discriminative patterns that appear at different time scales and positions. Methods that perform well under these conditions often trade transparency or computational efficiency for accuracy. Therefore, developing approaches that are simultaneously accurate, lightweight, and transparent remains a central goal for the field.

Existing TSC methods can be grouped into three families with complementary strengths and weaknesses. *Feature-based* approaches transform series into vector representations using statistical descriptors^3^, spectral coefficients from discrete Fourier or wavelet transforms (DFT/DWT)^4^, or model-based parameters such as ARIMA coefficients^5^. Shapelet-based methods^6,7^ identify short discriminative subsequences that produce human-interpretable decision rules; however, exhaustive searches over lengths and positions are costly and fixed-length shapelets may miss multiscale patterns. *Distance-based* approaches classify via pairwise similarity measures, most notably dynamic time warping (DTW)^8^, which is resilient to local temporal distortions but scales poorly and lacks explicit feature representations. *Learning-based* methods, including ensembles^9^ and deep neural networks^2,10,11^, learn complex representations directly from data. They often achieve state-of-the-art accuracy but typically require large labeled datasets, extensive hyperparameter tuning, and offer reduced interpretability.

High-performance benchmarks that blend or extend these paradigms have also emerged. ROCKET^12^ applies thousands of random convolutional kernels to each series and summarizes each kernel’s output using two simple statistics—the proportion of positive values (PPV) and the maximum activation (MAX). These features are then fed into a linear classifier, achieving competitive accuracy at low computational cost. Hydra^13^ extends ROCKET with multi-kernel ensembles to refine the accuracy–efficiency trade-off, while HIVE-COTE^14,15^ hierarchically combines shapelet-based, spectral, distance-based, and convolutional classifiers through weighted voting. These methods set strong baselines on the UCR Time Series Classification Archive^16,17^ but often demand substantial computational resources and offer limited built-in interpretability. In parallel, research on multiscale representation learning^18,19^ and interpretability evaluation^20,21^ has gained prominence. Despite these advances, many high-performing models still rely on heavy hyperparameter tuning or on post-hoc explanations whose faithfulness can be uncertain.

Against this backdrop, we previously proposed the linear law-based transformation (LLT)^22–24^ as a lightweight, transparent alternative. LLT uses time-delay embedding and spectral decomposition to derive near-invariant linear combinations (“laws”) from training series and then projects new series onto these laws, yielding a feature space in which classes are more linearly separable. Intuitively, the law vector is the direction along which the embedded sequence changes least relative to a symmetric embedding-derived (Hankel-type) matrix *S* (similarly to Jakovác et al.^25,26^); in spectral terms, it is an eigenvector associated with the eigenvalue of minimal magnitude of *S* (equivalently, a minimizer of $\Vert Sv\Vert$ or $v^\top S^2 v$). This minimal–absolute–eigenvalue (quadratic-form-on-$S^2$) view clarifies LLT’s robustness to common distortions. However, LLT’s reliance on a single embedding scale limits its ability to capture discriminative patterns that span diverse temporal extents.

We address this limitation with the *adaptive law-based transformation (ALT)*, a principled multiscale generalization of LLT. ALT systematically scans each series using variable-length, shifted windows. For every window, it performs an *l*-dimensional delay embedding and computes the eigenvector associated with the eigenvalue of minimal magnitude (i.e., the direction minimizing $\Vert Sv\Vert$ or $v^\top S^2 v$) as a shapelet law. These window-local laws are organized into class-specific dictionaries. At inference time, a new series is embedded under the same multiscale schedule and projected onto the dictionaries, producing features that quantify conformity to class-typical patterns across scales and positions. In effect, ALT retains the traceability and transparency of shapelet-style pipelines, preserves the robustness of distance-based approaches without pairwise alignment, and approaches ensemble-level accuracy with few hyperparameters and linear-algebraic efficiency.

Relative to fixed-length shapelets, ALT learns low-variance directions directly from embedded data and aggregates them by class, avoiding costly brute-force length/offset selection while remaining transparent. Compared with DTW-based classifiers, ALT yields an explicit representation that enables fast training and prediction with standard learners. Unlike deep or ensemble methods such as Hydra and HIVE-COTE, ALT relies on only a few tunable hyperparameters (window schedule, stride, embedding dimension) while producing structured and transparent representations. A key distinction relative to LLT is that ALT removes the fixed-scale bottleneck through a theoretically grounded minimum-$\Vert Sv\Vert$ (i.e., $S^2$-quadratic-form) multiscale, shifted-window mechanism and a consistent law-aggregation framework. In addition, ALT has the potential to operate effectively in streaming settings: once a new instance is classified, any law it reveals that proves unique or discriminative could be directly incorporated into the corresponding class dictionary. This would obviate costly retraining and allow the feature space to evolve online with the data.

The main *contribution* of this paper is ALT, a lightweight, multiscale alternative to existing TSC methods, supported by comprehensive evidence of its benefits. Using the *BasicMotions* dataset from the UCR repository, we introduce synthetic noise and evaluate how models without transformation, with LLT, and with ALT perform, with particular attention to their robustness to noise. We then extend the evaluation to a total of ten UCR datasets spanning diverse real-world domains, where we compare classifier performance with and without the ALT transformation. Beyond accuracy, ALT offers practical advantages: a small hyperparameter set, efficient training and inference, and explicit, inspectable representations (law vectors and class dictionaries). Finally, our evaluation protocol emphasizes statistical rigor, incorporating robustness checks under noise, $30\times$ repeated trials, and appropriate significance testing.

The remainder of this paper is organized as follows. “Methodology and experimental settings” introduces the proposed ALT method, describes the evaluation datasets and literature benchmarks, and outlines the experimental protocol. “Results and discussion” reports and discusses the experimental findings. Finally, “Conclusion and future work” concludes the paper and highlights avenues for future research. The full set of experimental results is presented in the Supplementary Information for completeness and reproducibility.

## Methodology and experimental settings

### Adaptive law-based transformation and classification

Consider a multivariate time series dataset with instances indexed by $i=1,2,\dots ,\tau$, channels (sensors) by $j=1,2,\dots ,m$, and lengths $h_i$ that may vary across instances. Let $x^{i,j}_{1:h_i}$ denote channel *j* of instance *i*, and $y^i \in \{1,2,\dots ,c\}$ its class.

#### Multiscale windowing and embedding

ALT operates under a user-specified schedule of triplets $\mathcal {R}=\{(r,l,k)\}$ of positive integers, where *r* denotes the window length, *l* the embedding dimension, and *k* the window shift (stride). Each triplet satisfies

1 $$\begin{aligned} r = s\,(2l - 2) + 1, \qquad \text {for some integer } s \ge 1, \end{aligned}$$

ensuring that each window contributes exactly $2l-1$ evenly spaced samples with spacing $s=\frac{r-1}{2l-2}$. We require $l\ge 2$. For each training instance *i*, channel *j*, and start index $t=1,k+1,2k+1,\dots ,h_i-r+1$, we form an $l\times l$ symmetric Hankel-type embedding^27^

2 $$\begin{aligned} S^{i,j,t}_{r,l,k}[p,q] \;=\; x^{i,j}_{\,t + (p+q-2)s}, \qquad p,q=1,2,\dots ,l, \end{aligned}$$

so that $S^{i,j,t}_{r,l,k}=(S^{i,j,t}_{r,l,k})^\top$ by construction.

#### Law extraction (training)

For each $S^{i,j,t}_{r,l,k}$ let $\lambda _1,\dots ,\lambda _l$ denote the eigenvalues of $S^{i,j,t}_{r,l,k}$, and let $v_1,\dots ,v_l$ be the corresponding eigenvectors. We select the eigenvector associated with the eigenvalue of minimal magnitude:

3 $$\begin{aligned} v^{i,j,t}_{r,l,k} \;=\; v_{\,\arg \min _{q\in \{1,\dots ,l\}} |\lambda _q|}. \end{aligned}$$

These *l*-dimensional vectors are referred to as the *shapelet laws*. For each channel *j* and schedule element (*r*, *l*, *k*) we assemble a *class-labeled law dictionary*

4 $$\begin{aligned} P^{j}_{r,l,k} \;=\; \big [\, v^{i,j,t}_{r,l,k} \,\big ] \in \mathbb {R}^{\,l \times N_{r,l,k}^{(j)}}, \qquad \pi ^{j}_{r,l,k} \in \{1,2,\dots ,c\}^{\,N_{r,l,k}^{(j)}}, \end{aligned}$$

where each column inherits the class label $y^i$ of its source instance, and $N^{(j)}_{r,l,k}$ denotes the number of generated *shapelet laws*. Optionally, ALT performs an *internal* split of the training instances: a proportion $\rho \in (0,1]$—the law-training (LT) ratio—is used solely to build the class-labeled law dictionaries, while the remaining training instances are transformed by ALT and used to fit the downstream classifier (see “Experimental settings” for the global train–test protocol). The collection $\{(P^{j}_{r,l,k},\pi ^{j}_{r,l,k})\}$ over all *j* and $(r,l,k)\in \mathcal {R}$ constitutes the trained model.

#### Linear responses (transformation)

Given a new length-*H* instance $z^j_{1:H}$ (one per channel *j*), and a fixed (*r*, *l*, *k*) with spacing *s*, ALT creates $o=\left\lfloor \dfrac{H-(l-1)s-1}{k}\right\rfloor + 1$ length-*l* *row embeddings* per channel:

5 $$\begin{aligned} A^{j}_{u,q} \;=\; z^j_{\,(u-1)k + (q-1)s+1}, \qquad u=1,2,\dots ,o,\; q=1,2,\dots ,l. \end{aligned}$$

Stacking channels yields per-channel matrices and *law responses* by right-multiplying with the corresponding dictionary:

6 $$\begin{aligned} M^{j}_{u,\alpha } \;=\; \sum _{q=1}^{l} A^{j}_{u,q}\, P^{j}_{r,l,k}[q,\alpha ], \end{aligned}$$

producing $M^{j}\in \mathbb {R}^{\,o \times N^{(j)}_{r,l,k}}$ for each channel *j*, which we keep separate until class-wise pooling.

#### Class-wise pooling and feature extraction

For each class *c* we retain the columns of $M^{j}$ whose laws originated from class *c* according to $\pi ^{j}_{r,l,k}$. We then square the responses (energy) and apply *two-stage pooling*:

1. *Across laws (columns):* for a chosen percentile $q\in [0,1]$, compute the *q*-quantile (or mean) along the law dimension, yielding an *o*-length sequence per channel.
2. *Across windows (rows):* aggregate the resulting sequence(s) via statistics such as mean, variance, excess kurtosis, or higher-order central moments.

For each (*r*, *l*, *k*), class *c*, channel *j*, and extraction method, this yields one scalar feature. Concatenating over all schedule elements yields a feature vector of size $|\mathcal {R}| \times c \times m \times n$, where *n* denotes the number of feature extraction methods. Together, these steps define the feature extraction function $\Phi (\cdot )$, which maps any new instance to its fixed-length feature vector representation.

#### Classifier training and evaluation

ALT is model-agnostic: the resulting features can be used with standard classifiers such as *k*-nearest neighbors, support vector machines, or ensembles. In our experiments we tune hyperparameters on training data and report out-of-sample accuracy and runtime.

#### Practical considerations

ALT accommodates variable-length time series provided that each chosen window length satisfies $r \le \min _i h_i$ and the structural constraint $r=s(2l-2)+1$. For inference, we also require $r\le H$ for each processed instance. Selecting eigenvectors according to the eigenvalue of minimal magnitude provides a stable definition of law vectors for symmetric embeddings and aligns with the minimum-$\Vert Sv\Vert$ (equivalently, $v^\top S^2 v$) interpretation. In practice, maintaining separate law dictionaries per channel and per schedule element facilitates modularity and analysis. Post-training pruning or selection of laws may further reduce redundancy and enhance transparency; we leave this as a direction for future research (see Section 4). Formal robustness results for the stability of minimum-eigenvalue law vectors under perturbations, including explicit error bounds for noise and misalignment, are provided in the Supplementary Information.

For completeness, Algorithm 1 provides a concise pseudocode summary of the complete training, transformation, and feature-extraction pipeline.

**Algorithm 1:**
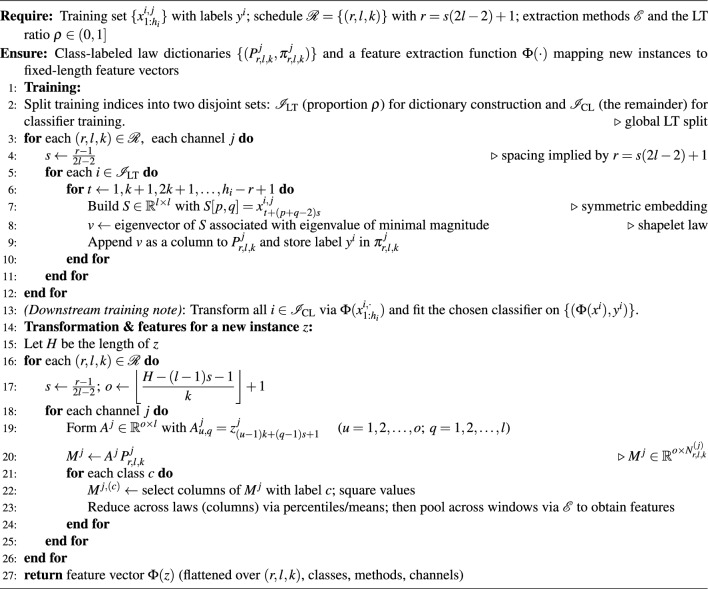
ALT training, transformation, and feature extraction

### Employed data

We evaluate ALT on ten widely used datasets from the UCR Time Series Classification Archive^16,17^, chosen to reflect standard benchmarks that capture core TSC challenges observed in practice, including noise and non-stationarity, local temporal misalignment, and multiscale or shifted motifs. These datasets cover both univariate and multivariate settings, short and long sequences, binary and multiclass problems, and diverse domains (motion, spectroscopy, biomedicine, and industrial). Table 1 summarizes the datasets; all are accessible at https://www.timeseriesclassification.com (retrieved: October 6, 2025).

**Table 1:** Datasets used in this study.

Dataset	Classes	Channels	Train	Test	Length	Domain	Description
BasicMotions	4	6	40	40	100	Motion	Motion sensor data from four activities performed by participants
Coffee	2	1	28	28	286	Spectroscopy	Spectrographs of two coffee bean types to distinguish between them
Epilepsy	4	3	137	138	207	Biomedical	Tri-axial accelerometer data from four tasks, including mimicking a seizure
Epilepsy2	2	1	80	11420	178	Biomedical	Single-channel EEG measurements to determine seizure occurrence
FordA	2	1	1320	3601	500	Industrial	Automotive engine noise measurements used to detect symptoms
FordB	2	1	810	3636	500	Industrial	Variant of FordA focusing on different engine symptoms
GunPoint1	2	1	50	150	150	Motion	X-axis hand motion data for “Gun-Draw” vs. “Point” actions
GunPoint2	2	1	135	316	150	Motion	GunPoint variant distinguishing participants from different age groups
GunPoint3	2	1	135	316	150	Motion	GunPoint variant distinguishing male vs. female participants
GunPoint4	2	1	135	316	150	Motion	GunPoint variant distinguishing old vs. young participants

All datasets are from the UCR Time Series Classification Archive^16,17^, accessible at https://www.timeseriesclassification.com (retrieved: October 6, 2025). Original names for the GunPoint variants: (1) GunPoint; (2) GunPointAgeSpan; (3) GunPointMaleVersusFemale; (4) GunPointOldVersusYoung.

### Literature benchmarks

For the benchmark datasets, we compare ALT against baseline models trained directly on the raw series without transformation, results obtained using our earlier LLT method, and reported test accuracies of state-of-the-art methods from the literature. These literature benchmarks are summarized in Table 2.

**Table 2:** Literature benchmarks.

Dataset	Test accuracy (%)	Reference	Method
BasicMotions	95.3–100.0	^28^	DTWD, ROCKET, CIF, HIVE-COTE
Coffee	96.0–100.0	^29^	Random Forest, ROCKET, MiniROCKET, MultiROCKET
Coffee	78.6–100.0	^30^	Raw-ResNet, FoldCount-1NN, TimeAxisArea-1NN, DWT-1NN
Epilepsy	96.3–100.0	^28^	DTWD, ROCKET, CIF, HIVE-COTE
Epilepsy	95.7–97.1	^31^	Debiased contrastive learning with weak supervision
Epilepsy	85.0–99.0	^32^	CNN
Epilepsy2	89.4–100.0	^33^	Multi-scaled embedding for large-scale time-series pretraining
FordA	96.8–100.0	^34^	Lightweight attention networks
FordA	79.3–86.4	^35^	LB-SimTSC (Similarity-aware graph neural network)
FordA	49.0–95.0	^29^	Random Forest, ROCKET, MiniROCKET, MultiROCKET
FordA	74.54–95.6	^36^	LSRSC (Centered Kernel Alignment)
FordA	56.7–93.6	^30^	Raw-ResNet, FoldCount-1NN, TimeAxisArea-1NN, DWT-1NN
FordA	53.4–71.3	^37^	Residual reservoir computing neural networks
FordA	89.0	^38^	Convolutional neural networks
FordA	96.5	^39^	Shapelet transform
FordA	50.6–90.9	^40^	Time-series/class-aware temporal and contextual contrasting
FordB	92.9–100.0	^34^	Lightweight attention networks
FordB	49.0–83.0	^29^	Random Forest, ROCKET, MiniROCKET, MultiROCKET
FordB	63.8–83.1	^36^	LSRSC (Centered Kernel Alignment)
FordB	53.1–81.7	^30^	Raw-ResNet, FoldCount-1NN, TimeAxisArea-1NN, DWT-1NN
FordB	51.9–56.4	^37^	Residual reservoir computing neural networks
FordB	70.0	^38^	Convolutional neural networks
FordB	91.5	^39^	Shapelet transform
FordB	50.9–88.2	^40^	Time-series/class-aware temporal and contextual contrasting
GunPoint1	85.0–100.0	^29^	Random Forest, ROCKET, MiniROCKET, MultiROCKET
GunPoint1	68.0–99.0	^30^	Raw-ResNet, FoldCount-1NN, TimeAxisArea-1NN, DWT-1NN
GunPoint2	57.0–100.0	^29^	Random Forest, ROCKET, MiniROCKET, MultiROCKET
GunPoint3	68.0–100.0	^29^	Random Forest, ROCKET, MiniROCKET, MultiROCKET
GunPoint4	88.0–100.0	^29^	Random Forest, ROCKET, MiniROCKET, MultiROCKET

All datasets are from the UCR Time Series Classification Archive^16,17^, accessible at https://www.timeseriesclassification.com (retrieved: October 6, 2025). Original names for the GunPoint variants: (1) GunPoint; (2) GunPointAgeSpan; (3) GunPointMaleVersusFemale; (4) GunPointOldVersusYoung.

### Experimental settings

We conducted two complementary experiments to evaluate ALT. The *first experiment* assessed robustness to noise on the *BasicMotions* dataset by adding zero-mean Gaussian noise as multipliers of standard normal noise (${0,1,\dots ,20} \cdot \mathcal {N}(0,1)$), yielding 21 perturbation levels from no noise to the highest distortion. At each noise level, models without transformation, with LLT, and with ALT were trained using k-nearest neighbors (KNN)^41^ and support vector machines (SVMs)^42^ and evaluated over 30 independent repetitions to ensure stable results. The *second experiment* extended the evaluation to ten datasets from the UCR repository (see Table 1), comparing ALT against raw baselines using both KNN and SVM, again with 30 repetitions per dataset. For additional comparison, we also trained both optimizable feed-forward neural networks and the ROCKET classifier directly on the raw time-series data.

Except for the neural networks and the ROCKET model—where we applied 500 and 30 iterations of Bayesian hyperparameter optimization, respectively, with cross-validation but without repetitions due to computational cost—all other models were tuned using 30 iterations of Bayesian optimization with five-fold cross-validation repeated 30 times. For the neural networks, five-fold cross-validation was used; for ROCKET, the number of folds was dataset-specific due to dataset size limitations: two-fold for *BasicMotions*, three-fold for *Coffee*, four-fold for the *GunPoint*, and five-fold for all remaining datasets. The hyperparameters of ALT ($r, l, k$) were optimized to maximize classification accuracy, and multiple statistical indicator pairs were evaluated during feature extraction. When ALT is used, we also apply the internal LT split defined in “Adaptive law-based transformation and classification”: only a $\rho$ fraction of the training instances contributes to dictionary construction, and the remaining instances are used to train the classifier on ALT features. In our experiments, $\rho$ is fixed per dataset (see Table 3) and not tuned.

From the law–response matrix *M*, we first reduced across laws (columns) (mean or 5th percentile), and then aggregated across windows (rows) (variance together with the third- and fourth-order central moments). For the neural networks, the ROCKET model, and for the first experiment on *BasicMotions*, we retained the original train–test split provided by the UCR repository. In all other experiments, we followed the recommended train–test ratios but resampled new random splits at each repetition. The dataset-specific parameter configurations are provided in Table 3.

**Table 3:** Dataset-specific ALT parameter configurations and law-training (LT) ratio.

Dataset	LT ratio	Method	Used (*r*, *l*, *k*) values
BasicMotions	0.25	Mean–mean, $5{\text {th}}$ percentile–$4{\text {th}}$ moment	(53, 27, 1)
Coffee	0.25	$5{\text {th}}$ percentile–mean, $5{\text {th}}$ percentile–variance	(3, 2, 1), (91, 10, 1), (101, 11, 1)
Epilepsy	0.25	Mean–mean	(29, 15, 1), (69, 35, 1), (89, 45, 1), (149, 75, 1), (169, 85, 1), (189, 95, 1)
Epilepsy2	0.25	$5{\text {th}}$ percentile–mean, $5{\text {th}}$ percentile–variance	(19, 10, 1), (29, 15, 1)
FordA	0.20	$5{\text {th}}$ percentile–mean	(23, 12, 1), (29, 15, 1), (85, 43, 1), (95, 48, 1), (205, 103, 1)
FordB	0.50	$5{\text {th}}$ percentile–mean	(19, 10, 1), (39, 20, 1), (129, 65, 1), (139, 70, 1), (159, 80, 1), (169, 85, 1),
			(179, 90, 1), (199, 100, 1), (209, 105, 1), (275, 138, 1)
GunPoint1	0.20	$5{\text {th}}$ percentile–mean, $5{\text {th}}$ percentile–variance	(13, 3, 1), (29, 8, 1), (41, 6, 1), (51, 6, 1), (91, 10, 1)
GunPoint2	0.50	$5{\text {th}}$ percentile–mean, $5{\text {th}}$ percentile–variance	(19, 4, 1), (31, 4, 1), (29, 3, 1), (141, 11, 1), (73, 10, 1)
GunPoint3	0.20	Mean–mean, $5{\text {th}}$ percentile–mean	(3, 2, 1), (19, 10, 1), (39, 20, 1), (109, 55, 1)
GunPoint4	0.50	Mean–mean	(3, 2, 1)

The law-training (LT) ratio denotes the fraction of training instances used to construct the law dictionaries. The remaining training instances (if any) are transformed via ALT and used for fitting the downstream classifier. The method column indicates a two-stage pooling: first aggregation across laws within each window (e.g., mean or $5{\text {th}}$ percentile), then aggregation across windows (e.g., mean, variance, excess kurtosis, or $4{\text {th}}$ central moment). All datasets are from the UCR Time Series Classification Archive^16,17^, accessible at https://www.timeseriesclassification.com (retrieved: October 6, 2025). Original names for the GunPoint variants: (1) GunPoint; (2) GunPointAgeSpan; (3) GunPointMaleVersusFemale; (4) GunPointOldVersusYoung.

## Results and discussion

To motivate our evaluation, we first demonstrate that ALT is able to extract linearly separable features from the *BasicMotions* dataset. We then use this dataset to study the robustness of the method under synthetic noise. Finally, we present benchmark results across ten datasets from the UCR repository.

### Linearly separable feature extraction

**Fig. 1:**
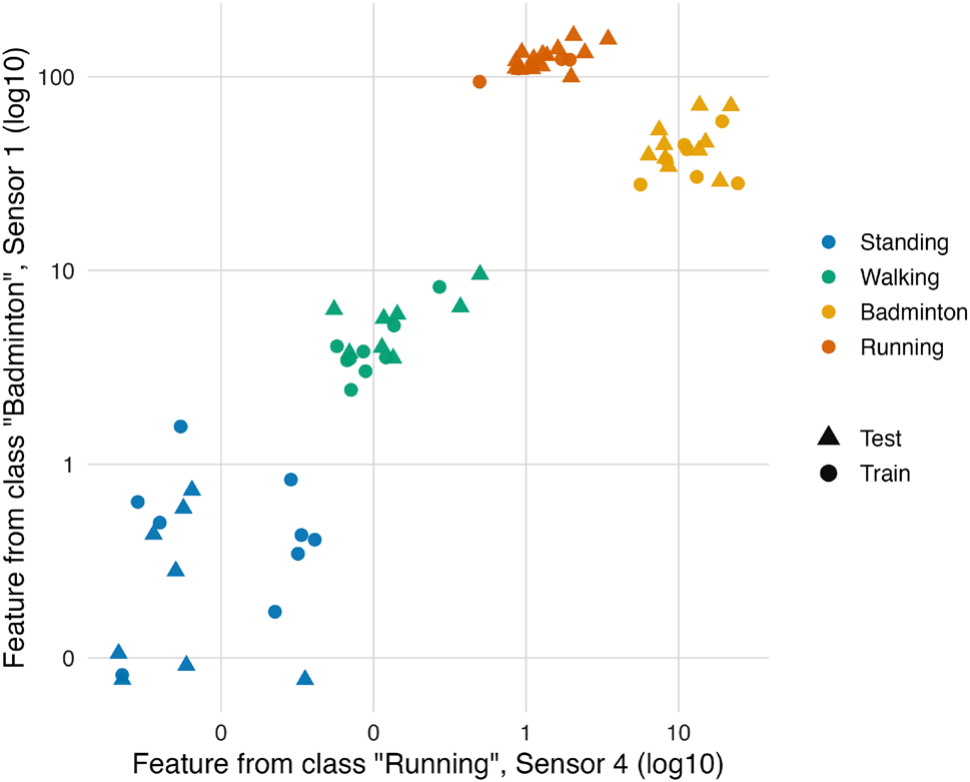
Two randomly chosen ALT features on *BasicMotions*: Running (Sensor 4) on the *x*-axis and Badminton (Sensor 1) on the *y*-axis (log–log). Points are colored by class; circles are train, triangles are test.

ALT’s behavior is illustrated through a single two-dimensional projection of its feature space on *BasicMotions*, which comprises four activities (Standing, Walking, Badminton, Running) recorded on six sensors. Using a mean–mean pooling configuration, ALT produces a compact representation per series; from the resulting feature vector, we select two coordinates at random: one feature derived from laws extracted from instances of the Running class on Sensor 4 (horizontal axis), and one feature derived from laws extracted from instances of the Badminton class on Sensor 1 (vertical axis). The resulting scatter plot is shown in Fig. 1.

Although this is only a two-feature view, the four classes form clean, well-separated clusters with small within-class scatter and clear margins. This reflects ALT’s design, which extends the LLT principle to a multiscale setting with shifted windows and two-stage pooling. While LLT emphasizes minimum-$\Vert Sv\Vert$ directions at a single scale, ALT collects responses across many local embeddings and then pools them, so that repeated alignments with class-typical patterns yield stable, discriminative feature values. The effect is that axes behave like class-informative coordinates, supporting nearly linear separation of the clusters. This example illustrates how ALT, through its multiscale construction, turns raw multichannel signals into compact and discriminative features that simplify downstream classification and analysis.

### Robustness to noise

**Fig. 2:**
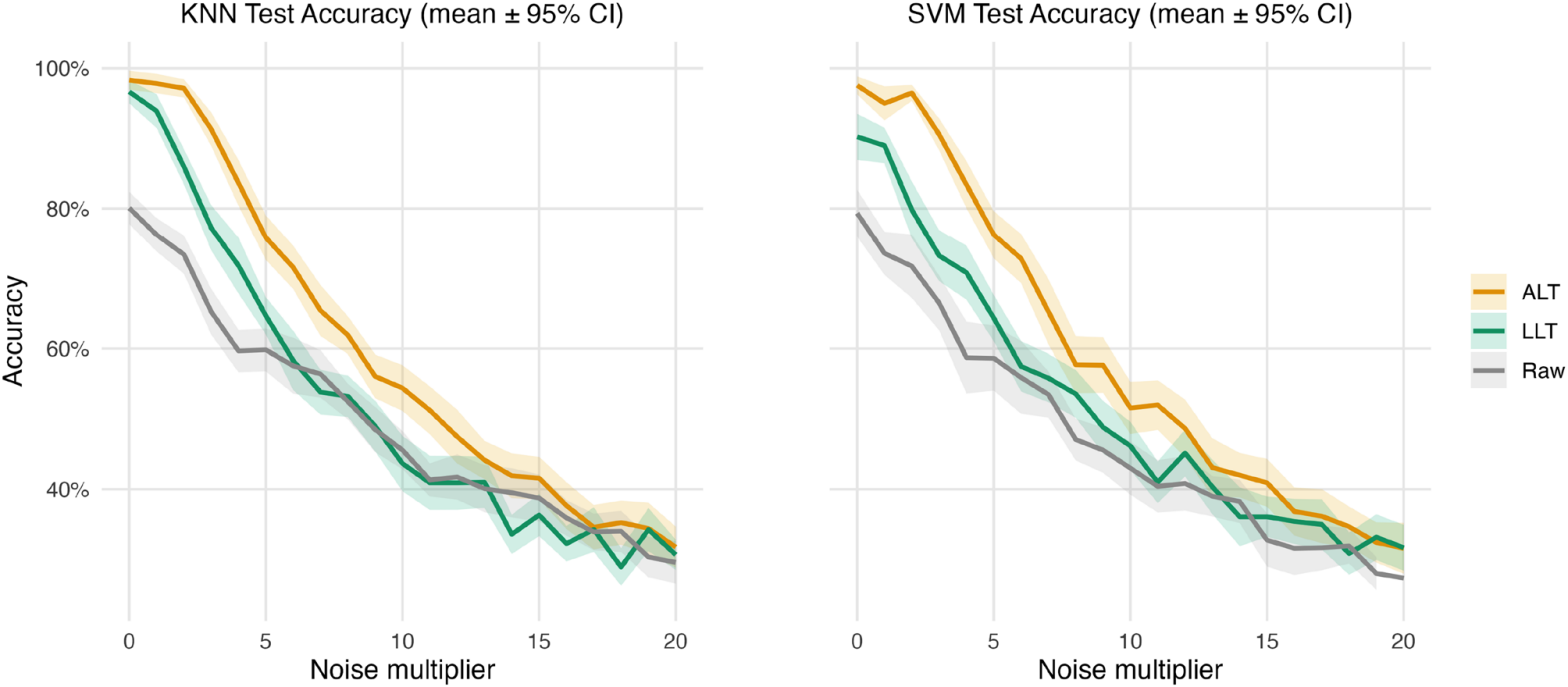
Test accuracy under increasing Gaussian noise on *BasicMotions*. Curves show mean accuracy with $\pm 95\%$ confidence intervals over 30 runs for KNN (left) and SVM (right), trained on ALT features (orange), LLT features (green), and raw inputs (gray). Noise levels correspond to multipliers of standard normal noise added to the series.

Figure 2 shows how test accuracy on *BasicMotions* degrades as Gaussian noise is added to the input series. The *x*-axis indicates the noise multiplier applied to standard normal noise ($\text {multiplier} \cdot \mathcal {N}(0,1)$), where a value of 0 denotes no noise and 20 the highest perturbation level. Results are reported for KNN and SVM classifiers trained on the original series (Raw), after LLT, and after ALT. Lines represent mean accuracy across 30 runs, with shaded ribbons denoting $\pm 95\%$ confidence intervals.

Across the full sweep of noise multipliers, ALT maintains the highest accuracy for both KNN and SVM at low–to–moderate noise levels (roughly 0–15), with LLT typically in between and the raw baseline generally lowest overall. At multiplier $=0$, ALT starts near 95–$100\%$, LLT is around 90–$97\%$, while Raw is around $80\%$. As noise increases, all three curves decline and the gaps narrow; LLT remains between ALT and Raw for most of the range. By the highest noise levels ($\approx 18$–20), the curves converge to roughly 27–$35\%$ and their 95% confidence intervals largely overlap; small crossings appear, indicating that the methods are effectively indistinguishable once the signal is overwhelmed by noise.

The confidence intervals confirm that ALT’s advantage is not a product of variability: its mean accuracy remains above LLT and Raw across most noise levels for both classifiers, with convergence and occasional crossings only at the highest noise multipliers. Full numerical results (means and standard deviations across the 30 runs at each noise level) are provided in Supplementary Table S1.

### Performance on benchmark datasets

**Fig. 3:**
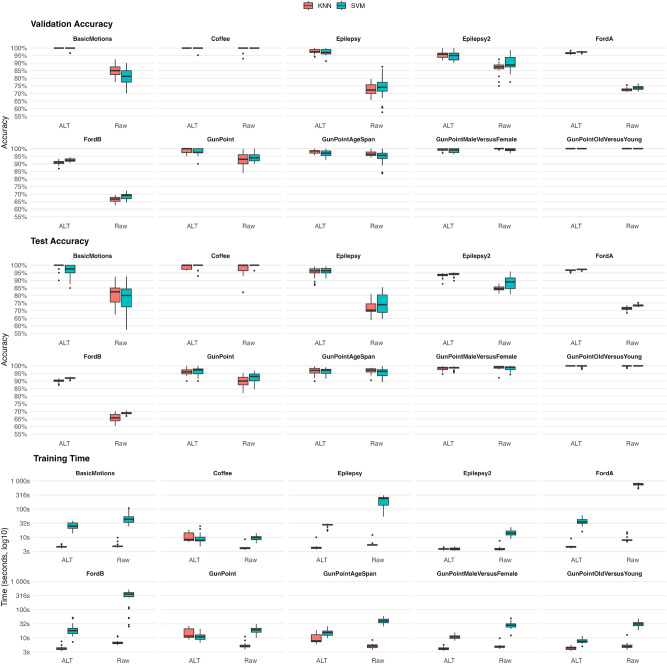
Validation accuracy (top), test accuracy (middle), and total training time (bottom) for KNN and SVM trained on ALT features (ALT) and original time series (Raw) across the benchmark datasets. Boxes show the distribution over 30 repetitions; central lines denote medians; whiskers extend to $\pm 1.5\times$ IQR.

Figure 3 summarizes validation accuracy, test accuracy, and total model training time (excluding ALT preprocessing) for KNN and SVM classifiers trained either on the original time series (without transformation) or on ALT features across the ten UCR datasets. Each boxplot aggregates $30\times$ repeated train–test resampling as specified in “Experimental settings”.

Across datasets, ALT improves or matches the median test accuracy for both classifiers; on saturated tasks (e.g., *Coffee*, *GunPointOldVersusYoung*), performance is already near 100% on the raw series, leaving no room for improvement. For *GunPointAgeSpan*, ALT yields a negligible change (KNN: –0.20 pp; SVM: + 0.80 pp). For *GunPointMaleVersusFemale*, ALT is slightly below the original representation (KNN: –0.70 pp, significant; SVM: –0.40 pp). Pooling all tasks, the macro-median gain is +7.6 pp for KNN and +4.8 pp for SVM. Clear advantages are observed on *BasicMotions*, *Epilepsy*, *Epilepsy2*, other *GunPoint* variants, and the large industrial datasets *FordA* and *FordB*. On *FordA* and *FordB*, ALT exceeds the original representation by +23.9–24.6 pp (medians across KNN/SVM), while also reducing training time substantially (median savings of approximately KNN: 3.3 s and 2.6 s; SVM: 717.5 s and 340.6 s).

Significance was assessed per dataset–classifier pair using paired Wilcoxon signed-rank tests over the 30 repetitions (ALT vs. original), with Holm–Bonferroni correction across the ten datasets at the 0.05 level, following Demšar^43^. On test accuracy, excluding the two saturated cases with $\ge$99% accuracy (*Coffee*, *GunPointOldVersusYoung*), ALT is significantly better on 6/8 datasets with SVM, and with KNN it is significantly better on 6/8, significantly worse on 1/8 (*GunPointMaleVersusFemale*), and not significant on 1/8 (*GunPointAgeSpan*). Validation-accuracy results are similar (again excluding the two saturated cases): KNN shows significant differences on 7/8 datasets (6 better, 1 worse: *GunPointMaleVersusFemale*), while SVM is significantly better on 7/8 and not significant on 1/8 (*GunPointMaleVersusFemale*). For *GunPointMaleVersusFemale* with KNN, the small negative difference remains significant after correction (Holm-adjusted $p=0.0205$). Notably, the gains on *FordA* and *FordB* are also highly significant, with very large effect sizes clearly favoring ALT.

Training-time distributions show that ALT’s preprocessing has limited wall-clock overhead for KNN and yields consistent speed-ups for SVM. Median total training time spans 3.8–11.7 s for KNN and 3.9–35.3 s for SVM (ALT), except for the original *FordA/B* with SVM, where several-hundred-second outliers occur. On the largest tasks, ALT reduces end-to-end SVM training time by 340.6–717.5 s while improving accuracy; however, this comes in addition to the one-off ALT feature transformation step, which required a median of 2.3–18.8 s across the simpler benchmarks and 77.4 s, 931.2 s, and 3220.2 s on *Epilepsy*, *FordA*, and *FordB*, respectively. While this preprocessing can be time-consuming on the largest datasets, training on the resulting law-based features is substantially faster than on the raw series. In streaming scenarios, newly observed laws can be incorporated directly at virtually no cost.

Overall, ALT delivers robust accuracy gains on most datasets—often large on the more challenging tasks—while preserving or improving training time. Where performance is already saturated (e.g., *Coffee*, *GunPointOldVersusYoung*), ALT maintains parity. The isolated negative cases (*GunPointAgeSpan*, *GunPointMaleVersusFemale*) are small in magnitude, with only one reaching significance, and do not affect the aggregate trend. Detailed per-dataset statistics for validation/test accuracy and training time (corresponding to Fig. 3) are reported in Supplementary Table S2. Supplementary Table S3 presents the full significance-testing results for all dataset–classifier pairs, and Supplementary Table S4 reports the ALT transformation times for the training and test splits.

For additional context, we also compared ALT’s performance with that of optimizable feed-forward neural networks and the ROCKET classifier, both trained directly on the raw time-series data. Table 4 summarizes their validation/test accuracies and training times across the same datasets.

**Table 4:** Comparison of classification performance on raw time series datasets using feed-forward neural networks and the ROCKET method.

Dataset	Feed-forward neural networks			ROCKET		
	Val. acc. (%)	Test acc. (%)	Time (s)	Val. acc. (%)	Test acc. (%)	Time (s)
BasicMotions	72.5	87.5	1941.7	90.0	95.0	265.9
Coffee	100.0	100.0	1105.7	100.0	100.0	419.2
Epilepsy	65.7	67.4	2745.3	94.3	90.6	781.3
Epilepsy2	80.0	89.9	1248.9	90.0	91.7	830.0
FordA	72.7	72.0	7347.9	93.2	90.5	963.6
FordB	63.0	66.0	5211.7	95.6	89.3	1146.2
GunPoint1	98.0	94.0	1380.7	91.7	82.7	644.8
GunPoint2	96.3	98.1	2023.2	100.0	98.4	685.6
GunPoint3	99.3	99.7	1513.2	96.0	99.4	662.3
GunPoint4	100.0	100.0	866.5	98.6	96.5	499.3

Results were obtained using Bayesian hyperparameter optimization with cross-validation. The feed-forward neural networks were trained with 500 optimization iterations, and ROCKET with 30 iterations. For the neural networks, five-fold cross-validation was used; for ROCKET, the number of folds was dataset-specific: two-fold for *BasicMotions*, three-fold for *Coffee*, four-fold for the *GunPoint*, and five-fold for all remaining datasets. All datasets are from the UCR Time Series Classification Archive^16,17^, accessible at https://www.timeseriesclassification.com (retrieved: October 6, 2025).

Relative to feed-forward neural networks and the ROCKET model trained directly on raw series (Table 4), ALT achieves substantially higher accuracy on noisy and complex datasets. For example, on *Epilepsy*, test accuracy rises from 67.4% (NNs) and 90.6% (ROCKET) to 96.4% (ALT-KNN and ALT-SVM); on *FordA*, from 72.0% and 90.5% to 97.3% (ALT-SVM); and on *FordB*, from 66.0% and 89.3% to 92.0% (ALT-SVM). Training times likewise shrink from thousands of seconds for neural networks (e.g., 7347.9 s on *FordA*) or several hundred seconds for ROCKET (e.g., 963.6 s on *FordA*) to only a few tens of seconds ($\approx$35 s).

Both neural networks and ROCKET achieve perfect or near-perfect accuracy on clean, low-variability datasets such as *Coffee* and *GunPointOldVersusYoung*, but their performance deteriorates on more difficult benchmarks where ALT remains robust. This contrast highlights ALT’s efficiency–robustness trade-off: strong accuracy on the hardest datasets, without the heavy computational burden of deep learning or large convolutional feature banks.

When set against published literature (Table 2), ALT is competitive though not universally state-of-the-art. On saturated benchmarks like *Coffee* and the *GunPoint* variants, ensemble pipelines (e.g., HIVE-COTE) and strong baselines (e.g., ROCKET, attention-based networks) already achieve near-perfect accuracy, leaving little headroom. By contrast, on industrial-scale, noisy datasets such as *FordA* and *FordB*, ALT delivers large and statistically significant gains over raw baselines, neural networks, and ROCKET—narrowing the gap to the strongest ensembles. For instance, ALT-SVM achieves 97.3% on *FordA* and 92.0% on *FordB*, approaching the ranges reported for advanced ensembles (96.5–100% and 91.5–100%, respectively).

In this sense, ALT occupies a middle ground: not always the top performer in absolute accuracy, but offering a distinctive balance of transparency, efficiency, and robustness. Moreover, it can also serve as a feature generator within ensemble pipelines, where its noise-robust features could further strengthen models such as HIVE-COTE.

### Computational complexity and resource usage

Let *N* denote the total number of training instances and $N_{\textrm{LT}}=\rho N$ the subset used for constructing the class-labeled law dictionaries (“Adaptive law-based transformation and classification”). Each instance *i* has *m* channels and length $h_i$, and ALT operates under a user-defined multiscale schedule $\mathcal {R}=\{(r,l,k)\}$, where *r* is the window length, *l* the embedding dimension, and *k* the stride (shift). For instance *i*, the number of windows processed under a given (*r*, *l*, *k*) is

7 $$\begin{aligned} w_i = \left\lfloor \frac{h_i - r}{k} \right\rfloor + 1. \end{aligned}$$

We define $L_{\max } = \max _{(r,l,k)\in \mathcal {R}} l$ and $W_{\max } = \max _i w_i$ as upper bounds on embedding dimension and number of training windows, respectively. Let $N_{\textrm{laws}} = \sum _{j,(r,l,k)} N^{(j)}_{r,l,k}$ denote the total number of stored law vectors. Note that $w_i$ counts symmetric training windows of length *r*, whereas $o=\left\lfloor \frac{H-(l-1)s-1}{k}\right\rfloor +1$ counts row embeddings used at inference time with span $(l-1)s+1$.

For each training instance $i \in \mathcal {I}_{\textrm{LT}}$, channel *j*, and schedule element (*r*, *l*, *k*), ALT forms $w_i$ symmetric $l \times l$ delay-embedding matrices $S^{i,j,t}_{r,l,k}$ and extracts the eigenvector $v^{i,j,t}_{r,l,k}$ associated with the eigenvalue of minimal magnitude. Constructing each *S* costs $O(l^2)$ and computing a dense eigendecomposition costs $O(l^3)$, with small constants as *l* is typically modest. Summing over all law-training instances and schedule elements gives

8 $$\begin{aligned} T_{\text {train,ALT}} = O\!\left( N_{\textrm{LT}}\, m \sum _{(r,l,k)\in \mathcal {R}} \mathbb {E}[w_i]\,(l^2 + l^3)\right) = O\!\left( \rho N\, m\, |\mathcal {R}|\, W_{\max }\, L_{\max }^3\right) . \end{aligned}$$

This one-off preprocessing step dominates ALT’s total runtime on large datasets, while subsequent classifier training on the compact ALT feature vectors is typically fast.

At inference time, each new instance of length *H* is transformed by computing, for every $(r,l,k)\in \mathcal {R}$ and channel *j*, an $o\times l$ matrix $A^{j}_{r,l,k}$ of row embeddings and multiplying it by the corresponding law dictionary $P^{j}_{r,l,k}$ of size $l\times N^{(j)}_{r,l,k}$. The resulting law–response matrix $M^{j}_{r,l,k}=A^{j}_{r,l,k} P^{j}_{r,l,k}$ requires $O(o\, l\, N^{(j)}_{r,l,k})$ operations. Summing over all channels and schedule elements gives

9 $$\begin{aligned} T_{\text {infer,ALT}} = O\!\left( \sum _{j=1}^{m}\sum _{(r,l,k)\in \mathcal {R}} o\, l\, N^{(j)}_{r,l,k}\right) = O\!\left( o\, L_{\max }\, N_{\textrm{laws}}\right) , \end{aligned}$$

The subsequent pooling of responses across laws and windows (percentile or moment statistics) is negligible compared with matrix multiplications. The memory footprint of the class-labeled dictionaries is

10 $$\begin{aligned} O\!\left( \sum _{j,(r,l,k)} l\, N^{(j)}_{r,l,k}\right) = O\!\left( L_{\max }\, N_{\textrm{laws}}\right) , \end{aligned}$$

which scales approximately linearly with $\rho$, *m*, $|\mathcal {R}|$, and the average number of windows per instance ($\approx W_{\max }$). This makes $N_{\textrm{laws}}$ directly tunable via the LT ratio, schedule density, or pruning of redundant laws.

ROCKET applies *k* random convolutions to each series and computes two statistics (PPV and MAX) per kernel, resulting in $\tilde{O}(kH)$ cost per instance and 2*k* features. In contrast, ALT concentrates most of its computational cost in the dictionary construction phase, with $O(L_{\max }^3)$ dependence on the embedding dimension. After this one-time preprocessing, ALT produces deterministic, structured feature vectors whose dimensionality is independent of the series length *H* and scales as $O\!\big (|\mathcal {R}|\, m\, c\, n\big )$, where *n* is the number of pooled statistics per (*r*, *l*, *k*), channel, and class. This design leads to substantially faster training of standard classifiers on ALT features, particularly for SVMs and other nonparametric learners.

Empirical runtime observations align with these theoretical bounds (see Supplementary Table S4). ALT preprocessing required 2.3–18.8 s on smaller benchmarks and 77.4 s, 931.2 s, and 3220.2 s on *Epilepsy*, *FordA*, and *FordB*, respectively. These costs are amortized over cross-validation and repeated runs, while SVM training on ALT features was substantially faster than on raw series (median reductions of 717.5 s on *FordA* and 340.6 s on *FordB*). Consequently, total wall time becomes competitive in multi-trial evaluation or streaming contexts, where dictionaries are reused.

A detailed, side-by-side comparison of computational complexity, feature dimensionality, and downstream training requirements between ALT and ROCKET is summarized in Table 5.

**Table 5:** Computational complexity, feature dimensionality, and downstream training requirements of ALT compared with ROCKET.

Method	Preprocessing/feature generation	Feature Dim.	Downstream training
ALT	$O\!\left( \rho N\, m\, |\mathcal {R}|\, W_{\max }\, L_{\max }^3\right)$ (training) | $O\!\left( o\,L_{\max }\,N_{\textrm{laws}}\right)$ (inference)	$O\!\big (|\mathcal {R}|\, m\, c\, n\big )$	Standard (e.g., KNN/SVM) on compact features
ROCKET	$\tilde{O}(k\,H)$ per instance	2*k*	Ridge/logistic regression on 2*k* features

*c* denotes the number of classes and *n* denotes the number of pooled statistics per (*r*, *l*, *k*), channel, and class (e.g., mean, variance, excess kurtosis, selected percentiles). ALT pools each schedule–channel combination to a small number of summary statistics, yielding a compact representation independent of sequence length *H*. For ROCKET, *k* is the number of random convolutional kernels (typically $k=10{,}000$), producing 2*k* features via the proportion of positive values (PPV) and maximum activation (MAX) per kernel.

ALT’s runtime and memory usage can be adjusted via simple design choices: reducing $|\mathcal {R}|$ or *l*, increasing stride *k* to decrease $W_{\max }$, lowering $\rho$ to shrink the dictionary size, or pruning near-duplicate laws. Each control scales $N_{\textrm{laws}}$ approximately linearly, allowing predictable trade-offs between computational cost, memory footprint, and representation richness.

## Conclusion and future work

This work introduced Adaptive Law-Based Transformation (ALT), a lightweight, transparent representation for time series classification that generalizes LLT by scanning sequences with a multiscale, shifted-window schedule and extracting minimum-$\Vert Sv\Vert$ (i.e., $S^2$-quadratic-form) “shapelet laws” from symmetric delay embeddings. The resulting class-labeled law dictionaries, combined with simple percentile–moment pooling, produce fixed-length features that standard learners can exploit while retaining transparent structure.

Across experiments, ALT consistently demonstrated robustness and efficiency. On *BasicMotions* under additive Gaussian noise, accuracy decayed more slowly with ALT than with LLT or raw inputs, with LLT typically between ALT and Raw and occasional crossings at higher noise levels (Fig. 2). Across ten UCR datasets, ALT features improved median validation and test accuracy for both KNN and SVM on most tasks, with particularly large margins on long and noisy industrial series such as *FordA* and *FordB*; by contrast, differences on *Coffee* were small (within about $\pm 1$ pp) and not reliably significant after correction (Fig. 3). In terms of practicality, cross-validation dominated wall-clock time, so ALT’s preprocessing introduced modest overhead on small datasets (2.3–18.8 s), around 1.3 min on *Epilepsy* (77.4 s), and longer runtimes on the largest industrial datasets (*FordA*: 931.2 s, *FordB*: 3220.2 s).

The current formulation still has limitations. Although the schedule (*r*, *l*, *k*) is low-dimensional, selecting it per dataset can require nontrivial tuning. Law dictionaries may contain redundancy or become memory-heavy under dense schedules or very long sequences. Pooling choices (percentiles across laws and moments across windows) are fixed rather than learned, and cross-channel dependencies are not modeled explicitly during law extraction. In addition, preprocessing can be time-consuming on the largest datasets, while training and streaming updates remain fast.

Looking ahead, several extensions can further strengthen ALT: data-driven discovery of (*r*, *l*, *k*) via structured Bayesian optimization or differentiable relaxations to learn scales and strides end-to-end; redundancy-aware dictionary pruning (e.g., submodular selection, sparsity penalties, or information criteria) with accuracy–memory guarantees; multi-channel law extraction using block-Hankel or tensor embeddings to capture cross-sensor structure; learnable pooling where percentile levels and aggregation statistics are parameterized and tuned by cross-validation or bilevel optimization; online/streaming variants with budgeted dictionary updates and drift detection to admit new laws without full retraining; theoretical analysis including perturbation bounds for minimum-eigenvector laws in Hankel embeddings and generalization guarantees under additive noise and local misalignment; and applications beyond classification to anomaly detection (law-conformity scores), segmentation (law change-points), and regression (law-weighted predictors).

Finally, ALT could also serve as a building block within ensemble pipelines. Its transparent, noise-robust features are complementary to convolutional or shapelet-based representations, and integration into methods such as HIVE-COTE could potentially enhance both accuracy and transparency. These directions aim to make ALT more adaptive, scalable, and theoretically grounded while preserving its core strengths of transparency and efficiency.

## Supplementary Information

Supplementary Information.

## Data Availability

This study uses ten real-world datasets from the UCR Time Series Classification Archive, available at https://www.timeseriesclassification.com (retrieved: October 6, 2025). Detailed descriptions of these datasets are provided in the study. For access to processed data subsets, contact the corresponding author.
