# Supplementary material for: Adaptive law-based feature representation for time series classification
Source: Sci Rep. 2025 Nov 25;15:41775. doi: 10.1038/s41598-025-25667-0 (PMC12647884; doi:10.1038/s41598-025-25667-0)
Supplement: Supplementary file 1 — Supplementary Information. [file 41598_2025_25667_MOESM1_ESM.pdf]

Supplementary Information for "Adaptive Law-Based Feature Representation for Time Series Classification"

Marcell T. Kurbucz[1,*], Balázs Hajós[2,3], Balázs P. Halmos[4,3], Vince Á. Molnár[4,3], and Antal Jakovác[3,5]

[1]Institute for Global Prosperity, The Bartlett, University College London, 9-11 Endsleigh Gardens, London, WC1H 0EH, United Kingdom
[2]Faculty of Science, Eötvös Loránd University, 1/A Pázmány Péter Walkway, Budapest, 1117, Hungary
[3]Department of Computational Sciences, Wigner Research Centre for Physics, 29-33 Konkoly-Thege Miklós Street, Budapest, 1121, Hungary
[4]Faculty of Engineering and Natural Sciences, Tampere University, Kalevantie 4, Tampere, 33100, Finland
[5]Department of Statistics, Corvinus University of Budapest, 8 Fővám Square, Budapest, 1093, Hungary
[*]m.kurbucz@ucl.ac.uk

Contents

Theoretical robustness of ALT . . . 2
Robustness to noise (Experiment 1) . . . 4
Performance on benchmark datasets (Experiment 2) . . . 5
Significance testing (Experiment 2) . . . 6
ALT transformation times (Experiment 2) . . . 7

Theoretical robustness of ALT

This section formalizes the robustness of the Adaptive Law-Based Transformation (ALT). For each symmetric embedding $S \in \mathbb{R}^{l\times l}$, ALT selects the unit eigenvector that minimizes $v^\top S^2 v$ (equivalently, the eigenvector associated with the eigenvalue of smallest absolute value of S). We work with S^2 because it preserves eigenvectors while making the spectrum nonnegative, which enables a clean application of matrix perturbation theory.

Assumption S1 (Bounded perturbation and eigengap)**.** *Let $S \in \mathbb{R}^{l\times l}$ be symmetric and let the perturbed (noisy or misaligned) embedding be $S' = S + E$ with $\|E\|_2 \le \varepsilon$. Since both S and S' are symmetric by construction, E is symmetric as well. Let the eigenvalues of S be ordered by absolute value, $|\lambda_1| \le |\lambda_2| \le \cdots \le |\lambda_l|$. Define the eigengap on S^2 by*

$$\delta \;:=\; \min_{j\ge 2} \left|\lambda_j^2 - \lambda_1^2\right| \;>\; 0,$$

which under this ordering equals $\lambda_2^2 - \lambda_1^2$ and implies that the smallest squared eigenvalue is simple (one-dimensional target subspace).

Lemma S1 (Weyl-type bound for the squared embedding)**.** *For $S' = S + E$,*

$$(S')^2 - S^2 \;=\; SE + ES + E^2 \quad \textit{and} \quad \|(S')^2 - S^2\|_2 \;\le\; 2\,\|S\|_2\,\|E\|_2 + \|E\|_2^2.$$

This follows from standard perturbation identities together with Weyl-type eigenvalue bounds for Hermitian matrices[1,2].

Theorem S1 (Stability of the minimum-$\|Sv\|$ (ALT law) direction)**.** *Let $v_\star$ be the unit eigenvector of S minimizing $v^\top S^2 v$ (i.e., corresponding to $|\lambda_1|$), and let $\hat{v}_\star$ be the analogous vector for S'. Applying the Davis–Kahan sin–Θ theorem to S^2 and $(S')^2$ under Assumption S1, we have*

$$\sin\angle(\hat{v}_\star, v_\star) \;\le\; \frac{\|(S')^2 - S^2\|_2}{\delta} \;\le\; \frac{2\,\|S\|_2\,\varepsilon + \varepsilon^2}{\delta}.$$

In the linear regime, e.g. for $\varepsilon \le \min\{1,\ \delta/(4\|S\|_2)\}$, this simplifies to

$$\sin\angle(\hat{v}_\star, v_\star) \;\le\; \frac{2\,\|S\|_2}{\delta}\,\varepsilon,$$

by neglecting the ε^2 term. See[1,2] for statements of the Davis–Kahan sin–Θ theorem for Hermitian operators.

Corollary S1 (Error in ALT responses before pooling)**.** *Let $A \in \mathbb{R}^{o\times l}$ be the row-embedding matrix for a new instance, let the class-labeled dictionary be $P = [v^{(1)}\ \cdots\ v^{(N)}] \in \mathbb{R}^{l\times N}$, and define the (pre-pooling) response matrix $M = AP$. Under perturbations $A' = A + \Delta_A$, $P' = P + \Delta_P$,*

$$\|M' - M\|_2 \;\le\; \|A\|_2\,\|\Delta_P\|_2 \;+\; \|\Delta_A\|_2\,\|P\|_2 \;+\; \|\Delta_A\|_2\,\|\Delta_P\|_2.$$

With columnwise sign alignment of eigenvectors,

$$\|\,\Delta v^{(\alpha)}\,\|_2 \;=\; \|\,\hat{v}^{(\alpha)} - v^{(\alpha)}\,\|_2 \;=\; 2\sin\!\left(\tfrac{\theta_\alpha}{2}\right) \;\le\; 2\sin\theta_\alpha,$$

where $\theta_\alpha = \angle(\hat{v}^{(\alpha)}, v^{(\alpha)})$. Hence $\|\Delta P\|_2 \le \|\Delta P\|_F \le 2\sqrt{N}\,\max_\alpha \sin(\theta_\alpha/2)$, and by Theorem S1 the response error is $\mathcal{O}(\varepsilon)$ provided δ is not too small.

Misalignment as a structured perturbation. Suppose each channel is L-Lipschitz (or has bounded first difference $\le L$). A local index shift by Δt changes entries in the symmetric Hankel-like embedding by at most $L\Delta t$. Standard operator-norm estimates for such matrices give

$$\|E\|_2 \;\le\; C_l\,L\Delta t,$$

with $C_l = \mathcal{O}(l)$ (e.g., via $\|E\|_2 \le \|E\|_F \le l\max_{p,q}|E_{pq}|$). Substituting $\varepsilon = C_l L\Delta t$ into Theorem S1 yields a misalignment-to-rotation bound:

$$\sin\angle(\hat{v}_\star, v_\star) \;\lesssim\; \frac{2\|S\|_2\,C_l L\Delta t + (C_l L\Delta t)^2}{\delta}.$$

Notes on eigengap and selection rule. ALT chooses the minimizer of $v^\top S^2 v$, i.e., the eigenvector for the smallest λ^2. This coincides with the eigenvector for the smallest $|\lambda|$ of S, but degeneracy can occur if distinct eigenvalues of S have the same absolute value (e.g., λ and $-\lambda$). Assumption S1 ($\delta > 0$) excludes this case and ensures a one-dimensional target subspace.

Practical guidance. (i) Avoid (r,l) settings where $|\lambda_1| \approx |\lambda_2|$ (tiny δ); (ii) Normalizing windows so that $\|S\|_2$ is $\mathcal{O}(1)$ tightens the bound when ε is measured after the same normalization; (iii) Increasing l may enlarge δ but also increases cost—tune (r,l,k) with this trade-off in mind.

Illustrative example (2D case). Consider a 2D setting where points lie on the x-axis: $X_a = (x_a, 0)$ for $a = 1,\dots,N$, with zero-mean x_a. The (uncentered) covariance matrix is $\Sigma = X^\top X/N$, so $\Sigma = \begin{bmatrix} \mathbb{E}[x^2] & 0 \\ 0 & 0 \end{bmatrix}$ in expectation. Hence the eigenvectors are e_x and e_y, with eigenvalues $\lambda_+ = \mathbb{E}[x^2]$ and $\lambda_- = 0$; the latter reflects the (initially zero) spread in the y direction.

Add i.i.d. zero-mean noise with variance σ^2, independent across a and coordinates: $X'_a = (x_a + \xi_{xa},\, \xi_{ya})$. Let $\Sigma' = X'^\top X'/N$. In 2D, one can compute the perturbed smallest eigenvalue via $\lambda'_- = \det(\Sigma')/\lambda'_+$; for small perturbations $\lambda'_+ \approx \lambda_+$. A direct calculation gives

$$\mathbb{E}[\Sigma'_{11}] = \mathbb{E}[x^2] + \sigma^2, \quad \mathbb{E}[\Sigma'_{22}] = \sigma^2, \quad \mathbb{E}[\Sigma'_{12}] = 0,$$

so $\mathbb{E}[\lambda'_-] = \sigma^2$, as expected: the y-extension grows to the noise level.

For the eigenvector rotation, denote the small angle by $\delta\alpha$. Using the first-order formula $\delta\alpha \approx -\Sigma'_{12}/\lambda_+$, we obtain

$$\mathbb{E}[\delta\alpha] = 0, \qquad \mathbb{E}[\delta\alpha^2] \approx \frac{\mathrm{Var}(\Sigma'_{12})}{\lambda_+^2} = \frac{\sigma^2\,\mathbb{E}[x^2] + \sigma^4}{N\lambda_+^2}.$$

Thus the expected squared rotation decays as $1/N$ and scales linearly with σ^2 for small noise (dominant term $\approx \sigma^2/(N\lambda_+)$ when $\sigma^2 \ll \mathbb{E}[x^2]$ and $\lambda_+ \approx \mathbb{E}[x^2]$). This explains the stability observed for ALT. In higher dimensions, small rotations can be accompanied by mixing among near-zero eigenvectors; this does not affect ALT's invariances, since laws are identifiable up to an invertible reparameterization.

Analogy with PCA noise robustness studies. Since PCA and ALT rely on the same underlying mathematics of eigenvector and eigenvalue analysis, their theoretical robustness properties are closely aligned. The reasoning presented above could just as well be applied to the largest eigenvalues, in which case it would describe the classical robustness of PCA. Moreover, extensive literature exists on PCA's sensitivity to noise—see, for example,[3–5]. These results, with minor adaptations, can also be used to analyze the noise robustness of ALT.

Robustness to noise (Experiment 1)

Noise	ALT		LLT		Raw	
	Mean	SD	Mean	SD	Mean	SD
**KNN Test Accuracy**						
0	0.983	0.036	0.967	0.044	0.801	0.062
1	0.978	0.037	0.939	0.064	0.762	0.064
2	0.972	0.035	0.860	0.067	0.734	0.073
3	0.913	0.064	0.772	0.087	0.652	0.087
4	0.837	0.088	0.718	0.107	0.597	0.080
5	0.758	0.085	0.647	0.071	0.598	0.081
6	0.716	0.083	0.583	0.112	0.576	0.106
7	0.654	0.097	0.538	0.086	0.564	0.090
8	0.619	0.072	0.532	0.079	0.525	0.068
9	0.561	0.083	0.490	0.097	0.485	0.086
10	0.544	0.087	0.437	0.106	0.456	0.073
11	0.512	0.091	0.409	0.103	0.413	0.063
12	0.475	0.103	0.409	0.103	0.418	0.086
13	0.442	0.073	0.410	0.098	0.401	0.090
14	0.419	0.086	0.336	0.075	0.395	0.094
15	0.416	0.082	0.363	0.081	0.388	0.090
16	0.377	0.089	0.322	0.068	0.359	0.068
17	0.346	0.085	0.342	0.085	0.339	0.070
18	0.352	0.084	0.289	0.071	0.340	0.078
19	0.344	0.098	0.342	0.082	0.303	0.077
20	0.318	0.079	0.307	0.058	0.296	0.080
**SVM Test Accuracy**						
0	0.976	0.034	0.902	0.088	0.793	0.090
1	0.950	0.065	0.890	0.068	0.736	0.082
2	0.965	0.031	0.798	0.109	0.718	0.121
3	0.906	0.061	0.733	0.097	0.665	0.104
4	0.834	0.090	0.708	0.103	0.588	0.136
5	0.762	0.090	0.643	0.089	0.587	0.123
6	0.728	0.094	0.575	0.095	0.559	0.138
7	0.652	0.124	0.558	0.092	0.535	0.088
8	0.578	0.108	0.536	0.091	0.471	0.080
9	0.577	0.105	0.488	0.100	0.456	0.087
10	0.516	0.100	0.462	0.093	0.430	0.101
11	0.520	0.095	0.410	0.079	0.404	0.100
12	0.487	0.110	0.452	0.089	0.408	0.104
13	0.431	0.112	0.403	0.104	0.390	0.078
14	0.420	0.085	0.361	0.112	0.382	0.082
15	0.409	0.091	0.361	0.077	0.328	0.101
16	0.368	0.092	0.354	0.086	0.316	0.102
17	0.362	0.103	0.350	0.096	0.317	0.086
18	0.347	0.077	0.308	0.081	0.319	0.067
19	0.324	0.077	0.332	0.087	0.280	0.062
20	0.316	0.097	0.317	0.087	0.273	0.083

Table S1. Noise-test accuracy (mean and standard deviation, SD) for adaptive law-based transformation (ALT), linear law-based transformation (LLT), and original untransformed time series (Raw) using k-nearest neighbors (KNN) and linear support vector machine (SVM) classifiers, across noise multipliers 0–20. Gaussian noise scaled by the multiplier was added to the input, and results were averaged over 30 runs. The *BasicMotions* dataset is part of the UCR Time Series Classification Archive, available at `https://www.timeseriesclassification.com` (retrieved: October 6, 2025).

Performance on benchmark datasets (Experiment 2)

Dataset	ALT				Raw			
	KNN_med	KNN_SD	SVM_med	SVM_SD	KNN_med	KNN_SD	SVM_med	SVM_SD
**Validation Accuracy**								
BasicMotions	1.000	0.000	1.000	0.012	0.850	0.037	0.813	0.054
Coffee	1.000	0.000	1.000	0.018	1.000	0.016	1.000	0.000
Epilepsy	0.976	0.016	0.971	0.019	0.723	0.040	0.741	0.067
Epilepsy2	0.958	0.025	0.950	0.027	0.875	0.038	0.888	0.052
FordA	0.966	0.007	0.974	0.004	0.726	0.011	0.736	0.014
FordB	0.911	0.014	0.924	0.009	0.668	0.020	0.686	0.063
GunPoint1	1.000	0.016	0.975	0.023	0.930	0.041	0.940	0.030
GunPoint2	0.985	0.014	0.971	0.018	0.963	0.016	0.956	0.040
GunPoint3	0.991	0.009	0.991	0.013	1.000	0.003	0.993	0.007
GunPoint4	1.000	0.000	1.000	0.000	1.000	0.000	1.000	0.000
**Test Accuracy**								
BasicMotions	1.000	0.021	0.975	0.041	0.825	0.061	0.800	0.088
Coffee	1.000	0.016	1.000	0.021	1.000	0.047	1.000	0.007
Epilepsy	0.964	0.031	0.964	0.020	0.703	0.043	0.728	0.080
Epilepsy2	0.938	0.013	0.943	0.012	0.846	0.016	0.890	0.045
FordA	0.968	0.006	0.973	0.003	0.715	0.011	0.734	0.007
FordB	0.904	0.011	0.920	0.006	0.657	0.025	0.689	0.065
GunPoint1	0.960	0.020	0.973	0.025	0.900	0.039	0.930	0.032
GunPoint2	0.970	0.023	0.972	0.021	0.972	0.019	0.964	0.029
GunPoint3	0.987	0.012	0.987	0.010	0.994	0.015	0.991	0.017
GunPoint4	1.000	0.000	1.000	0.005	1.000	0.004	1.000	0.000
**Training Time (s)**								
BasicMotions	4.558	0.384	24.991	6.940	4.776	1.023	43.472	22.048
Coffee	8.417	4.115	7.830	4.246	4.121	0.852	9.774	1.982
Epilepsy	4.139	1.107	27.894	3.400	5.332	1.249	236.862	78.242
Epilepsy2	3.848	0.239	3.888	0.404	3.697	0.742	14.026	3.333
FordA	4.630	0.866	35.280	9.463	7.962	1.707	752.804	93.405
FordB	4.064	0.782	18.119	11.557	6.655	1.306	358.727	142.080
GunPoint1	11.712	6.364	10.909	3.380	5.066	1.382	19.229	4.787
GunPoint2	7.842	4.319	15.461	3.870	5.154	0.904	39.708	8.395
GunPoint3	4.123	0.482	10.935	1.658	4.816	0.984	28.088	7.607
GunPoint4	4.411	0.561	7.574	1.256	4.988	1.588	31.216	6.622

Table S2. Median (med) and standard deviation (SD) of validation accuracy, test accuracy, and training time (seconds) on adaptive law-based transformation (ALT) and original untransformed time series (Raw) using k-nearest neighbors (KNN) and linear support vector machine (SVM) classifiers. All datasets are from the UCR Time Series Classification Archive, accessible at https://www.timeseriesclassification.com (retrieved: October 6, 2025). Original names for the GunPoint variants: (1) GunPoint; (2) GunPointAgeSpan; (3) GunPointMaleVersusFemale; (4) GunPointOldVersusYoung.

Significance testing (Experiment 2)

Dataset	KNN				SVM			
	Diff	p_{Holm}	Sig	Cliff	Diff	p_{Holm}	Sig	Cliff
**Validation Accuracy**								
BasicMotions	+15.00 pp	1.39e-05	***	1.000	+18.75 pp	1.52e-05	***	1.000
Coffee	+0.00 pp	1.74e-01		0.100	+0.00 pp	7.36e-02		-0.167
Epilepsy	+25.31 pp	1.45e-05	***	1.000	+23.00 pp	1.52e-05	***	1.000
Epilepsy2	+8.33 pp	1.45e-05	***	0.989	+6.25 pp	1.35e-03	**	0.578
FordA	+23.98 pp	1.45e-05	***	1.000	+23.80 pp	1.52e-05	***	1.000
FordB	+24.32 pp	1.45e-05	***	1.000	+23.77 pp	1.52e-05	***	1.000
GunPoint1	+7.00 pp	1.45e-05	***	0.829	+3.50 pp	2.61e-03	**	0.567
GunPoint2	+2.23 pp	1.16e-03	**	0.587	+1.50 pp	1.45e-02	*	0.460
GunPoint3	-0.93 pp	6.72e-04	***	-0.520	-0.19 pp	7.36e-02		-0.231
GunPoint4	+0.00 pp	—		0.000	+0.00 pp	—		0.000
**Test Accuracy**								
BasicMotions	+17.50 pp	1.73e-05	***	0.998	+17.50 pp	1.82e-05	***	0.969
Coffee	+0.00 pp	1.00e+00		0.098	+0.00 pp	9.31e-02		-0.173
Epilepsy	+26.09 pp	1.73e-05	***	1.000	+23.55 pp	1.82e-05	***	1.000
Epilepsy2	+9.25 pp	1.73e-05	***	0.996	+5.35 pp	3.30e-04	***	0.580
FordA	+25.27 pp	1.73e-05	***	1.000	+23.88 pp	1.82e-05	***	1.000
FordB	+24.67 pp	1.73e-05	***	1.000	+23.07 pp	1.82e-05	***	1.000
GunPoint1	+6.00 pp	1.73e-05	***	0.888	+4.33 pp	1.49e-04	***	0.702
GunPoint2	-0.16 pp	1.00e+00		-0.024	+0.79 pp	5.79e-01		0.109
GunPoint3	-0.63 pp	2.05e-02	*	-0.414	-0.32 pp	9.82e-01		-0.144
GunPoint4	+0.00 pp	1.63e-01		0.167	+0.00 pp	5.24e-02		-0.267
**Training Time (s)**								
BasicMotions	-0.22 s	2.03e-02	*	-0.409	-18.48 s	1.83e-05	***	-0.833
Coffee	+4.30 s	1.83e-05	***	0.964	-1.94 s	1.75e-01		-0.324
Epilepsy	-1.19 s	1.83e-05	***	-0.936	-208.97 s	1.83e-05	***	-1.000
Epilepsy2	+0.15 s	4.97e-01		0.184	-10.14 s	1.83e-05	***	-1.000
FordA	-3.33 s	1.83e-05	***	-0.940	-717.52 s	1.83e-05	***	-1.000
FordB	-2.59 s	1.83e-05	***	-0.940	-340.61 s	1.83e-05	***	-0.967
GunPoint1	+6.65 s	1.83e-05	***	0.971	-8.32 s	1.83e-05	***	-0.816
GunPoint2	+2.69 s	1.83e-05	***	0.962	-24.25 s	1.83e-05	***	-0.998
GunPoint3	-0.69 s	2.42e-04	***	-0.689	-17.15 s	1.83e-05	***	-0.989
GunPoint4	-0.58 s	3.91e-04	***	-0.598	-23.64 s	1.83e-05	***	-1.000

Table S3. Significance summary for Table S2 (ALT vs Raw). Diff: Validation/Test—percentage points (pp), Time—seconds. p_{Holm}: Holm–Bonferroni–corrected p-value (paired Wilcoxon where applicable; two-sided). Sig: * < 0.05, ** < 0.01, *** < 0.001. Note: for time, the test was run on log-times; medians and Diff are reported in seconds. Cliff δ: effect size on the original scale. All datasets are from the UCR Time Series Classification Archive, accessible at https://www.timeseriesclassification.com (retrieved: October 6, 2025). Original names for the GunPoint variants: (1) GunPoint; (2) GunPointAgeSpan; (3) GunPointMaleVersusFemale; (4) GunPointOldVersusYoung.

ALT transformation times (Experiment 2)

Dataset	Training transformation time (s)	Test transformation time (s)
BasicMotions	2.254	0.172
Coffee	3.218	0.432
Epilepsy	77.445	2.824
Epilepsy2	5.060	115.617
FordA	931.208	1081.128
FordB	3220.196	1429.831
GunPoint1	4.722	3.084
GunPoint2	18.751	6.505
GunPoint3	12.385	4.919
GunPoint4	7.246	1.034

Table S4. Processing times (in seconds) for adaptive law-based transformation (ALT), measured separately for training and test transformations. All datasets are from the UCR Time Series Classification Archive, accessible at https://www.timeseriesclassification.com (retrieved: October 6, 2025). Original names for the GunPoint variants: (1) GunPoint; (2) GunPointAgeSpan; (3) GunPointMaleVersusFemale; (4) GunPointOldVersusYoung.

References

1. Stewart, G. W. & Sun, J.-g. *Matrix Perturbation Theory* (Academic Press, San Diego, 1990).

2. Bhatia, R. *Matrix analysis*, vol. 169 (Springer Science & Business Media, 2013).

3. Anderson, T. W. Asymptotic theory for principal component analysis. *Annals Math. Stat.* **34**, 122–148, DOI: 10.1214/aoms/1177704255 (1963).

4. Jolliffe, I. T. & Cadima, J. Principal component analysis: a review and recent developments. *Philos. Transactions Royal Soc. A: Math. Phys. Eng. Sci.* **374**, 20150202, DOI: 10.1098/rsta.2015.0202 (2016).

5. Candès, E. J., Li, X., Ma, Y. & Wright, J. Robust principal component analysis? *J. ACM* **58**, 1–37, DOI: 10.1145/1970392.1970395 (2011).
